# Secretory Profile of Adipose-Tissue-Derived Mesenchymal Stem Cells from Cats with Calicivirus-Positive Severe Chronic Gingivostomatitis

**DOI:** 10.3390/v14061146

**Published:** 2022-05-25

**Authors:** Antonio J. Villatoro, María del Carmen Martín-Astorga, Cristina Alcoholado, Liliya Kazantseva, Casimiro Cárdenas, Fernando Fariñas, José Becerra, Rick Visser

**Affiliations:** 1 Laboratory of Bioengineering and Tissue Regeneration, Department of Cell Biology, Genetics and Physiology, Biomedical Research Institute of Málaga (IBIMA), University of Málaga, 29071 Málaga, Spain; ajvillatoro@immunestem.com (A.J.V.); mcmartinastorga@gmail.com (M.d.C.M.-A.); alcoholado.c@gmail.com (C.A.); lkazantseva@bionand.es (L.K.); becerra@uma.es (J.B.); 2 Grupo Ynmun, Inmunología Clínica y Terapia Celular (IMMUNESTEM), 29071 Málaga, Spain; 3 Research Support Central Services (SCAI) of the University of Málaga, 29071 Málaga, Spain; ccg@uma.es; 4 Grupo Ynmun, Spanish Association for the Research in Immunological and Infectious Diseases, 29071 Málaga, Spain; farinas.inmunologia@gmail.com; 5 Biomedical Research Networking Center in Bioengineering, Biomaterials, and Nanomedicine (CIBER-BBN), 28029 Madrid, Spain

**Keywords:** cats, mesenchymal stem cells, secretome, feline chronic gingivostomatitis, immunoassay, ultra-high-performance liquid chromatography high-resolution mass spectrometry (UHPLC–HRMS), bioinformatics

## Abstract

The feline calicivirus (FCV) causes infections in cats all over the world and seems to be related to a broad variety of clinical presentations, such as feline chronic gingivostomatitis (FCGS), a severe oral pathology in cats. Although its etiopathogeny is largely unknown, FCV infection is likely to be a main predisposing factor for developing this pathology. During recent years, new strategies for treating FCGS have been proposed, based on the use of mesenchymal stem cells (MSC) and their regenerative and immunomodulatory properties. The main mechanism of action of MSC seems to be paracrine, due to the secretion of many biomolecules with different biological functions (secretome). Currently, several pathologies in humans have been shown to be related to functional alterations of the patient’s MSCs. However, the possible roles that altered MSCs might have in different diseases, including virus-mediated diseases, remain unknown. We have recently demonstrated that the exosomes produced by the adipose-tissue-derived MSCs (fAd-MSCs) from cats suffering from FCV-positive severe and refractory FCGS showed altered protein contents. Based on these findings, the goal of this work was to analyze the proteomic profile of the secretome produced by feline adipose-tissue-derived MSCs (fAd-MSCs) from FCV-positive patients with FCGS, in order to identify differences between them and to increase our knowledge of the etiopathogenesis of this disease. We used high-resolution mass spectrometry and functional enrichment analysis with Gene Ontology to compare the secretomes produced by the fAd-MSCs of healthy and calicivirus-positive FCGS cats. We found that the fAd-MSCs from cats with FCGS had an increased expression of pro-inflammatory cytokines and an altered proteomic profile compared to the secretome produced by cells from healthy cats. These findings help us gain insight on the roles of MSCs and their possible relation to FCGS, and may be useful for selecting specific biomarkers and for identifying new therapeutic targets.

## 1. Introduction

Feline calicivirus (FCV) is one of the main agents responsible for infection and disease in felines worldwide [1]. Although its infection is frequently subclinical, it seems to be a predisposing factor for a wide number of clinical manifestations, such as feline chronic gingivostomatitis (FCGS), which is one of the most severe oral pathologies in cats [2,3]. The estimated prevalence of this oral mucosal inflammatory pathology is between 0.7 and 12% among cat populations and is more frequently found in feline colonies [4,5]. The etiopathogenesis of FCGS is still not clear, although it is known to include a significant immune-mediated component that is associated with several factors. Among these, FCV infection seems to be one of the most important predisposing conditions of the pathology [6,7]. Currently, the proposed treatments for FCGS include dental extraction and different immunosuppressant drugs, which help in controlling the disease but fail to provide a cure in up to 30% of the patients. Hence, a large number of the patients become refractory [8].

During the last decade, some promising strategies using mesenchymal stem cells (MSCs) for the treatment of FCGS have emerged, based on the immunoregulatory and regenerative potential that these cells have [9,10]. However, it is currently assumed that the mechanism of action of the MSCs to maintain homeostasis and to modulate the responses of the immune system is mainly paracrine and relies on the production and release of a great variety of biomolecules with different biological functions. Altogether, these components secreted by the cells, which include growth factors, cytokines, and extracellular vesicles, among others, are known as the secretome [11,12,13]. In some human pathologies, such as lupus, type 2 diabetes, or metabolic syndrome, it has been shown that the MSCs of the patients are dysfunctional and may participate in the etiopathogenesis of the diseases [14,15,16,17]. However, in veterinary medicine, not much is known about the role that alterations of the MSCs might have on the development or progress of diseases, especially those caused by viral infections [18,19].

Recently, our group demonstrated that the exosomes produced by adipose-derived MSCs (fAd-MSCs) isolated from patients with FCV-positive severe and refractory FCGS contained an altered protein profile, which might be related to a dysfunctional biological effect [20]. However, it is still not known whether these alterations only affect the exosomal fraction of the secretome or also extend to other components. Therefore, our aim in this work was to analyze the soluble factors and the proteomic profile of the secretome of fAd-MSCs from patients with calicivirus-positive severe and refractory FCGS to detect any possible alterations related to the disease. This will lead to a better understanding of this pathology and the possibility of using autologous fAd-MSC for its treatment.

## 2. Materials and Methods

The protocols used in this study were approved by the Ethics Committee of the Andalusian Center for Nanomedicine and Biotechnology (BIONAND, Málaga, Spain). All procedures that affected the cats were carried out by veterinarians after the owners had given informed consent, approving the use of samples from their pets for the experiments included in this study.

### 2.1. Selection Criteria

The study included ten client-owned European shorthair cats. Five cats were selected as healthy controls and five were FCV-positive FCGS patients. The inclusion criteria for cats in the FCGS group were the following: unspayed or unneutered cats suffering from severe FCGS as confirmed by a biopsy, with all their molars extracted a minimum of six months earlier and not showing any clinical improvement after immunomodulatory therapies [21]. To discard other pathologies in these animals, a clinical exploration and biochemical and hematological tests were performed. However, conditions such as hyperglobulinemia, hyperproteinemia, and neutrophilia were accepted, since these have been previously shown to be related to chronic gingivostomatitis. Dental radiographs confirmed the lack of dental roots. Oropharyngeal samples of the selected cats were used to test them positive for FCV infection by RT-PCR. Blood samples were used for testing the cats negative for both the feline leukemia and immunodeficiency viruses (SNAP Combo FeLV/FIV, IDEXX Laboratories, Westbrook, ME, USA) [21]. The Stomatitis Disease Activity Index (SDAI) was used to determine how severe the FCGS of each cat was [20,22]. Only cats that scored at least 20 points out of 30 were selected for the FCGS group.

No pathologies were detected in any of the healthy control cats. Using the same clinical and laboratory tests, these animals were proven to be negative for the viral infections mentioned earlier.

None of the cats received any medication for at least 10 days prior to extracting the adipose tissue samples. For palliative purposes, only analgesic drugs were allowed for the cats with FCGS when necessary.

### 2.2. Isolation and Culturing of fAd-MSCs

The animals were sedated using midazolam (Midazolan B. Braun, Melsungen, Germany; 0.1 mg/kg, IV), ketamine (Imalgene, Merial, France; 10–20 mg/cat, IV), and butorphanol (Turbogesic, Zoetis, Madrid, Spain; 0.1 mg/kg IV) for clinical examinations that did not involve traumatic procedures and were not related to this study. Approximately 5 g of subcutaneous adipose tissue was taken from the inguinal area of each cat, maintaining the samples in Dulbecco’s modified Eagle medium (DMEM, Merck, Darmstadt, Germany) at 4 °C until they were transferred to the laboratory within 12 h.

Afterwards, fAd-MSCs from the donors were isolated, cultured, and characterized using protocols previously optimized in our laboratory [13,20,23]. Briefly, the samples were digested with collagenase type I (Merck, Darmstadt, Germany) and the cells were plated on T-175 flasks with DMEM containing 10% fetal bovine serum (FBS), L-glutamine (2.5 mM), penicillin (100 U/mL), streptomycin (100 µg/mL), and fungizone (1.25 µg/mL) (Merck, Darmstadt, Germany). The cells were maintained under standard culture conditions (i.e., 37 °C, 5% CO_2_, 95% relative humidity) and they were subcultured when confluency reached 80%. The cells were used at passage in all the further experiments. They were characterized using flow cytometry to demonstrate the expression of MSC markers and the absence of hematopoietic markers [24]. Finally, according to the guidelines of the International Society of Cellular Therapy, their capacity to differentiate into, osteogenic, chondrogenic, and adipogenic lineages was evaluated [13,24].

### 2.3. Production of Secretome from fAd-MSCs

The cells from each donor were cultured separately. Here, 1.5 × 10^6^ fAd-MSCs was cultured in T-175 flasks with 10% FBS, L-glutamine (2.5 mM), penicillin (100 U/mL), and streptomycin (100 µg/mL), changing the medium twice per week until the cells reached 80% confluency. At this point, the cultures were washed with PBS, then 20 mL of fresh, non-supplemented DMEM was added to each flask. After 24 h, the conditioned media were recovered and filtered through a 0.22 µm filter to eliminate cells and debris. The obtained secretomes were stored at −80 °C until used for characterization. The cells remaining in the flasks were detached with trypsin and quantified in a Neubauer chamber with trypan blue staining to determine viability.

### 2.4. Characterization of the fAd-MSC-Derived Secretomes by Immunoassay

To quantify the main cytokines and chemokines, the secretomes of the five healthy donors and those of the five FCGS patients were combined in two separate pools. The volume of secretome used from each individual donor was normalized according to the number of cells that had produced the secretome. The selected biomolecules were quantified using a MILLIPLEX^®^ Feline Cytokine/Chemokine Magnetic Bead Panel (Merck), applying Luminex xMAP technology. The following 19 molecules were analyzed: Fas, granulocyte-macrophage colony-stimulating factor (GM-CSF), FMS-like tyrosine kinase 3 ligand (Flt-3L), interferon-gamma (IFN-γ), interleukin 1β (IL-1β), IL-2, IL-4, IL-6, IL-8, IL-12, IL-13, IL-18, platelet-derived growth factor-BB (PDGF-BB), stromal cell-derived factor 1 (SDF-1), keratinocyte chemoattractant (KC), stem cell factor (SCF), monocyte chemoattractant protein-1 (MCP-1), and tumor necrosis factor-α (TNF-α), as well as regulated on activation, normal T cell expressed and secreted (RANTES). All the obtained values were normalized and finally expressed as picograms per milliliter and per 10^6^ cells.

### 2.5. Proteomic Profile of the Secretome by Liquid Chromatography High-Resolution Mass Spectrometry and Bioinformatic Analysis

Here, 5 mL secretome samples were lyophilized and subsequently reconstituted in 200 µL of milli-Q water, sonicated, and centrifuged at 16,000× *g* at 4 °C for 5 min. Afterwards, gel-assisted proteolysis was performed by entrapping the samples in a polyacrylamide gel matrix where they were reduced with dithiothreitol, alkylated with iodoacetamide, and digested with trypsin (Promega, Madison, WI, USA). Finally, the peptides were extracted from the gel and purified using a C18 ZipTip (Merck Millipore; Burlington, MA, USA) according to the manufacturer’s instructions.

The peptide samples were injected into an Easy nLC 1200 UHPLC system that was coupled to a hybrid linear trap quadrupole Orbitrap Q-Exactive HF-X mass spectrometer (Thermo Fisher Scientific; Waltham, MA, USA). The HPLC solvents used were 0.1% formic acid in water (solvent A) and 0.1% formic acid in 80% acetonitrile (solvent B). Samples were then automatically loaded onto a trap column (Acclaim PepMap 100, 75 μm × 2 cm, C18, 3 μm, 100 A, Thermo Fisher Scientific) using a flow rate of 20 µL min-1 and eluted onto a 50 cm analytical column (PepMap RSLC C18, 2 μm, 100 A, 75 μm × 50 cm, Thermo Fisher Scientific). The elution of the peptides from the analytical column was performed using a 120 min gradient ranging from 2% to 20% of solvent B, a 30 min gradient from 20% to 35% of solvent B, and finally to 95% solvent B for 15 min before re-equilibration to 2% solvent B at a constant flow rate of 300 nL min^−1^. The Tune 2.9 and Xcalibur 4.1 software (Thermo Fisher Scientific) were used to obtain the data. MS1 scans were performed from *m*/*z* 300 to 1750 at a resolution of 120,000. The 20 precursor ions that were most intense were isolated within a 1.2 *m*/*z* window using a data-dependent acquisition method and fragmented to obtain the corresponding MS/MS spectra. The fragment ions were generated in a higher-energy collisional dissociation (HCD) cell and detected at a resolution of 30,000 in an Orbitrap mass analyzer.

To analyze the raw data that were obtained, we used the Proteome DiscovererTM 2.4 platform (Thermo Fisher Scientific) with the SEQUEST^®^ HT engine using mass tolerances of 10 ppm and 0.02 Da for precursor and fragment ions, respectively. MS/MS spectra were searched against the NCBI protein database for *Felis catus*, version 2017.10.30 (43,896 sequences). The protein assignments were validated using the Percolator^®^ algorithm imposing a strict 1% false discovery rate (FDR) cut-off. The results were filtered out so that only those proteins that had at least two identified peptide sequences were accepted.

Label-free quantitation was implemented using the Minora feature in Proteome Discoverer^TM^ 2.4. The protein abundances were based on precursor intensities. A normalization was performed based on the amount of total peptide and the abundance ratio *p*-values were calculated using an ANOVA based on the abundances of individual proteins. The enrichment analyses for the biological process, molecular function, and cellular components of the identified proteins were performed according to the international standardized gene function classification system from Gene Ontology (http://www.geneontology.org/; accession date 16 April 2021) using the network-based annotation service from Proteome DiscovererTM 2.4.

Furthermore, we used STRING (v.11.5) (www.string-db.org; accession date 14 February 2022) to determine any possible interactions between differentially expressed proteins. Using sources such as the genomic context, experimental and co-expression data, as well as previous information, STRING detects both direct (physical) and indirect (functional) interactions between proteins. All STRING analyses were performed using the *Felis catus* database. Each node represents a protein, and each link represents a protein–protein interaction. The interactions included functional and physical associations with a medium confidence score (0.04).

### 2.6. Statistical Analysis

Prism 6.0c (ed. 2013, GraphPad Software Inc., San Diego, CA, USA) was used to perform statistical analyses. Student’s *t*-test was used to compare differences between means of the ELISA assay results. The following degrees of significance were established: *p* < 0.05 (*), *p* < 0.01 (**), and *p* < 0.001 (***). Data are represented as means ± SD.

## 3. Results

### 3.1. Selected Cats

We selected five heathy and five FCGS-positive European shorthair cats. Each group containing two females and three males. The FCGS group had an average weight of 3.4 ± 0.6 kg, an average age of 6.4 ± 3.7 years, and an average SDAI score of 22.4 ± 0.9. The healthy cats group had an average weight of 3.8 ± 0.24 kg and an age of 4.2 ± 1.3 years (Table 1).

### 3.2. Characterization of the fAd-MSCs

The fAd-MSCs isolated from all selected animals were found positive for the mesenchymal stem cell markers CD29, CD44, CD73, CD90, CD105, and MHC-I, and negative for the hematopoietic markers CD34, CD45, and MHC-II (Figure S1A). When induced appropriately, the cells differentiated to the three mesodermal lineages. The presence of fat drops stained with Oil Red O indicated adipogenic differentiation. Osteogenic differentiation was demonstrated by alizarin red staining of calcium salt deposits. Chondrogenic differentiation was evidenced by the cell’s ability to form micromasses that exhibited metachromasia when they were stained with toluidine blue (Figure S1B).

### 3.3. Characterization of the Secretomes by Immunoassay

**Figure 1:**
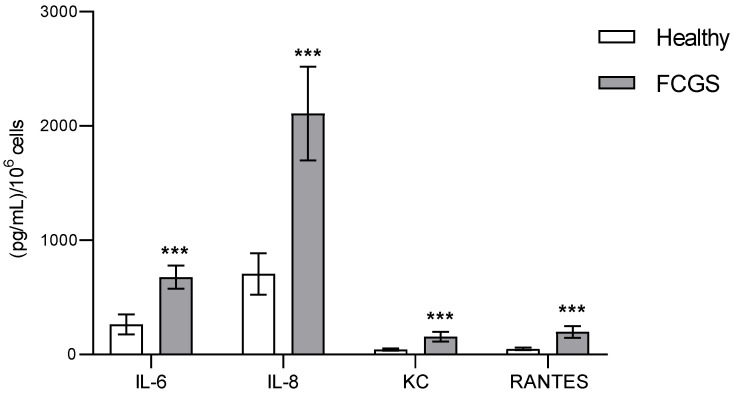
Cytokines detected in the fAd-MSC-derived secretomes from healthy and FCGS cats. The obtained values were normalized per 10^6^ cells. Note: *** *p* < 0.001.

From all 19 biomolecules analyzed via immunoassay, 15 had a concentration below the detection limit of the technique, as indicated by the manufacturer. The four analytes that could be detected (IL-6, IL-8, KC, and RANTES) were all pro-inflammatory and their concentrations were significantly higher in the diseased cats than in the healthy ones (Figure 1).

### 3.4. Proteomic Analysis of the fAd-MSC-Derived Secretomes

**Figure 2:**
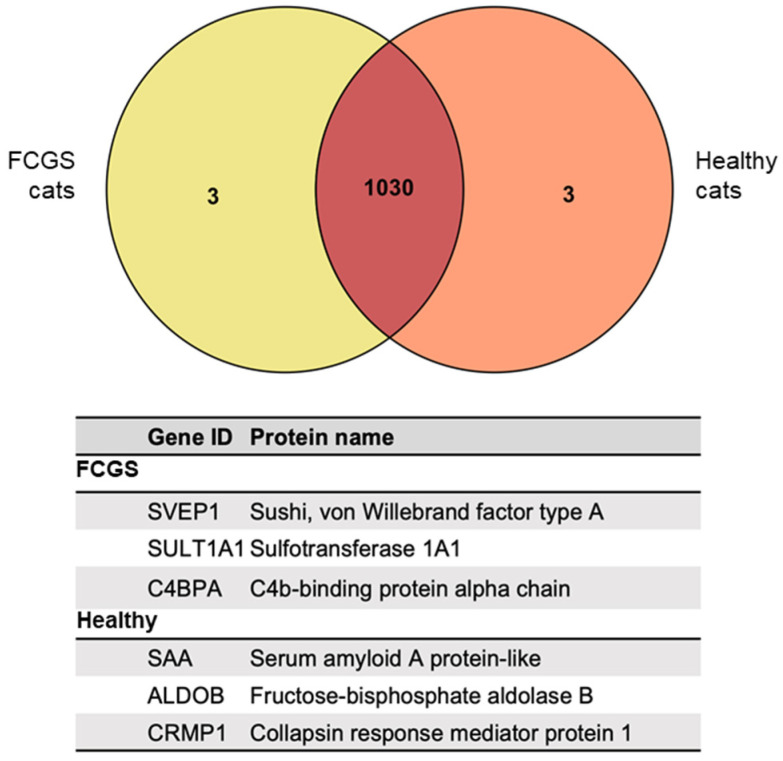
Venn diagram showing the proteins exclusively found in the secretome of healthy cats and those only found in FCGS-positive cats.

After performing the proteomic analysis using high-resolution mass spectrometry, and according to the *Felis catus* database, we identified 1030 proteins that were expressed in both groups of animals (Figure 2). The complete list of proteins is presented in Table S1. In addition, we identified three secretome-specific proteins that could only found in the secretome from the FCGS group (SVEP1, SULT1A1, and C4BPA), and three other proteins that were exclusively found in the secretome from the healthy donors (SAA, ALDOB, and CRMP1).

### 3.5. Functional Enrichment Analysis Based on Gene Ontology and STRING Database

**Figure 3:**
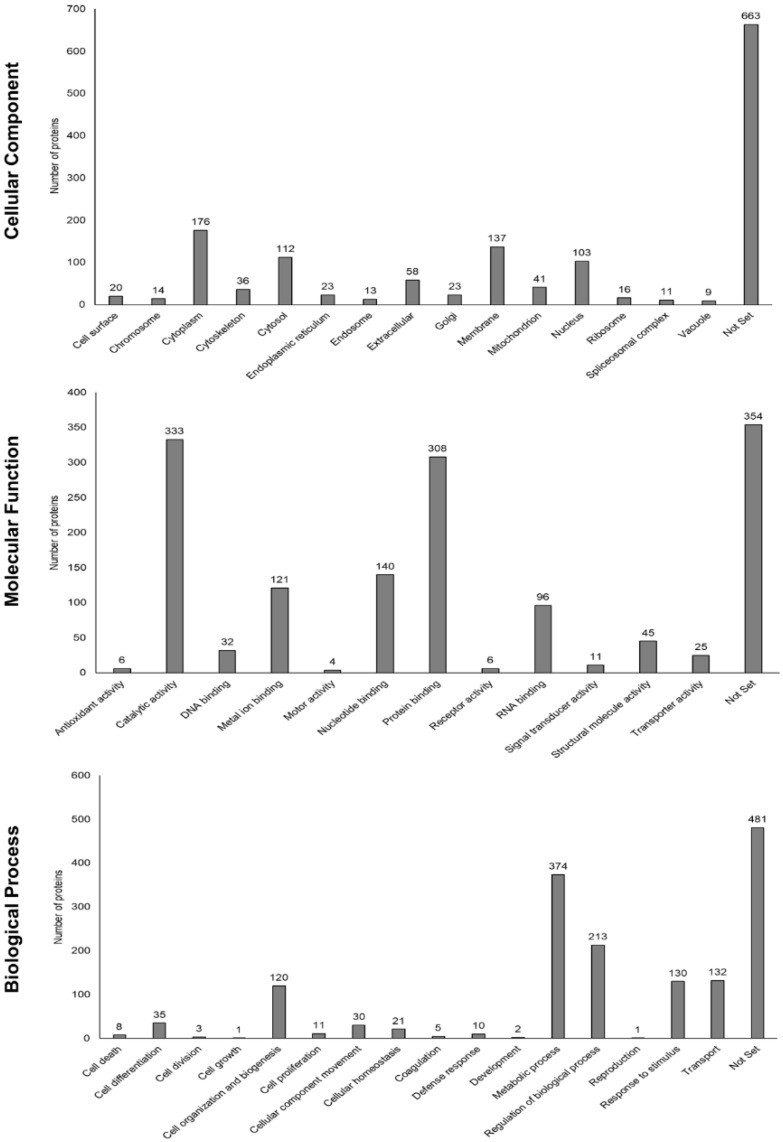
Gene Ontology annotations related to proteins identified within the secretomes produced by fAd-MSCs. A specific protein might be included in different subgroups. “Not set” denotes that these proteins could not be classified in any of the parameters described according to Gene Ontology (more information can be found in Table S1).

The *Felis catus* peptide database was used to perform a bioinformatic analysis. Each protein was functionally annotated based on Gene Ontology (GO) parameters (process, molecular function, or cellular component) (Figure 3).

The proteins that were found to be most abundant within each group of cats were classified as cytoplasmatic proteins (understood as the entire cell content, excluding the cell membrane and the nucleus but including other subcellular structures), followed by proteins localized in the membrane, the cytosol (the part of the cytoplasm not containing organelles but containing other particles, such as protein complexes), and nucleus proteins. Furthermore, the secretomes from both groups were enriched with proteins related to metabolic processes, the regulation of biological processes, transport, responses to stimuli, and cell organization and biogenesis. Based on the molecular function of the proteins that we found, the predominant protein fraction was related to catalytic activity, as well as binding to proteins, nucleotides, metal ions, and RNA, in this order. The full list of proteins, considering that a certain protein can be involved in different functions, is shown in Table S1.

### 3.6. Label-Free Quantification of the Secretome Proteomes

**Figure 4:**
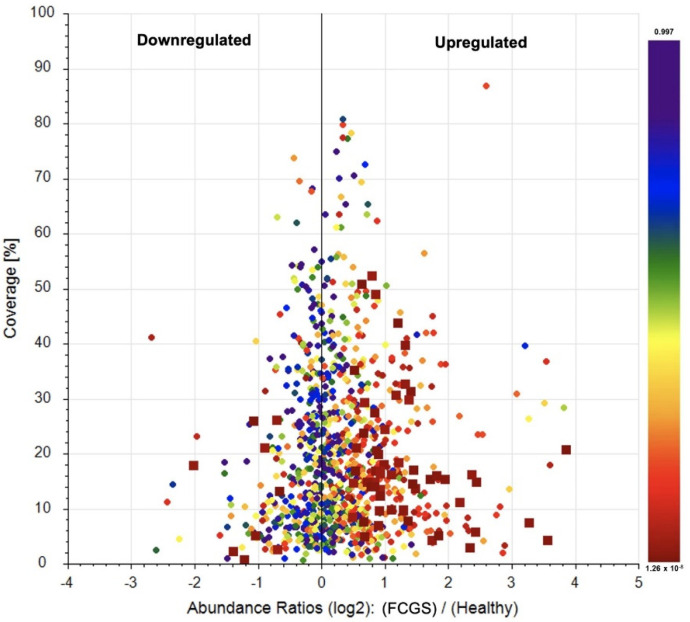
Proteins up- and downregulated in the FCGS cats. Scatter plots showing the percentages of protein sequence coverage vs. their Log2 fold change abundance values in the secretome from FCGS cats versus healthy donors. Proteins were ranked according to their *p*-values from red to blue color. A protein was considered down- or upregulated (red squares) when its fold-change value was <0.5 or >2, respectively, with a *p*-value < 0.05.

After we compared the protein profiles of the secretomes from both groups of cats, we could identify 56 proteins that were significantly (*p* < 0.05) upregulated in the FCGS group, while 9 proteins were significantly downregulated compared to the control group (Figure 4). These specific proteins are listed in Table S1.

### 3.7. Interactions between Differentially Expressed Proteins

**Figure 5:**
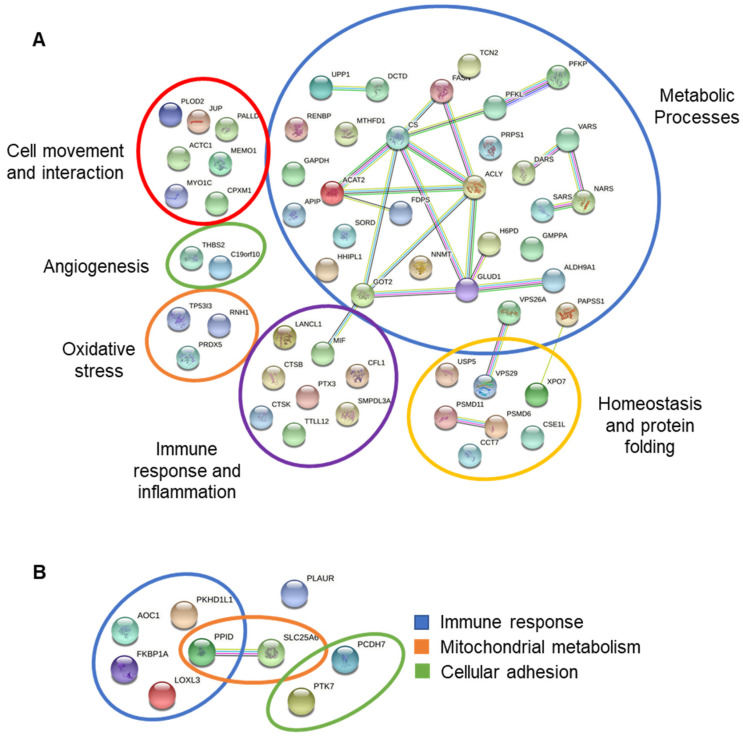
Upregulated (**A**) and downregulated (**B**) protein networks were identified in the fAd-MSC-derived secretome. Schematic representation of both known and predicted interactions between proteins according to the STRING database (v.11.5). Each node represents a protein, and each edge represents an interaction. Only those interactions with mean confidence scores (0.4) are represented. The interactions shown can be either physical or functional associations. The legend for the represented proteins can be found in Table S2.

Using STRING, the up and downregulated proteins found in the FCGS group compared to the control group were classified with a map of possible interactions and functions. Upregulated proteins were related to the metabolic process (50.9%), immune response and inflammation (14.5%), homeostasis and protein folding (12.75%), cell movement and interaction (12.75%), oxidative stress (5.45%), and angiogenesis (3.65%). On the other hand, downregulated proteins were related to immune response (55.5%), mitochondrial metabolism (22.2%), and cellular adhesion (22.2%) (Figure 5, Table S2).

## 4. Discussion

The knowledge that we have about the responses of MSCs against viral infections in clinical circumstances is still scarce, especially in feline veterinary [25]. In this work, we have been able to identify for the first time the parts of the biomolecules that form the secretome produced by fAd-MSCs from FCV-positive FCGS patients.

Using immunoassays, we found that 4 out of the 19 analytes that we tried to quantify had a concentration above the limit of detection of the test: IL-6, IL-8, KC, and RANTES. All these molecules have a pro-inflammatory effect, and they were all significantly more abundant in the samples derived from the FCGS cats than in those from healthy cats. IL-6 is a multifunctional cytokine produced by different cell types, which is involved in a great number of immune responses. It is considered a biomarker of systemic inflammation [26,27], and it has been previously reported that its concentration in plasma increases in refractory FCGS patients. IL-8, also known as CXCL8, is a chemokine with a high capacity to induce chemotaxis through the recruitment of neutrophils and their infiltration into the tissues, contributing to the elimination of pathogens. It also has a potent pro-angiogenic activity that induces venous endothelial proliferation [28]. Increased levels of IL-8 in plasma have been reported in cats suffering from feline idiopathic cystitis and feline osteoarthritis [29,30]. RANTES, also known as CCL5, is a chemotactic chemokine involved in leukocyte migration. It is also known to exert a beneficial pro-inflammatory role in viral infections [31]. Lastly, the keratinocyte chemoattractant (KC) is a chemotactic factor known to play an important role in the induction of systemic inflammation and tissue damage after traumatism or hemorrhage [32].

These changes observed in the secretion of factors towards a pro-inflammatory profile in the fAd-MSCs of FCV-positive FCGS patients are similar to those observed in MSCs from other species when suffering viral infections [25,33] or after experimental transfection with the virus in disease models [34]. It is, however, important to keep the limitations of the multiplex assay in mind regarding its sensitivity; other analytes might have remained undetected, as has happened in other studies on feline patients using this technique [27].

Proteomic analysis based on high-resolution mass spectrometry (HRMS) is a useful and powerful tool to characterize the protein profile of different biological samples. This technique, together with the *Felis catus* database, allowed us to identify 1030 proteins that were secreted by fAd-MSCs from both healthy and diseased cats. Three additional proteins (ALDOB, CRMP1, and SAA) were solely expressed by cells from the healthy cats, while three others (SVEP1, SULT1A1, and C4BPA) were only expressed by cells from the FCGS cats.

The fructose biphosphate aldolase (ALDOB) is an important enzyme that participates in glucose and fructose metabolism and is mainly localized in the liver, as well as in renal and intestinal cells [35]. It is known that metabolic reprogramming is closely related to epithelial–mesenchymal transition during tumor progression, where glycolysis plays an essential role [36]. ALDOB is important for the proliferation and invasion of cancer cells, and its overexpression is used as a prognosis biomarker for different adenocarcinomas in humans [37,38]. CRMP1 participates in axon guidance, neuronal growth cone collapse, and cell migration [39,40]. The serum amyloid A (SAA) is an acute phase protein, and it has been shown to increase its expression in cats during viral infections and inflammatory processes [41]. Sushi, von Willebrand factor type A, EGF, and pentraxin domain containing 1 (SVEP1) is an extracellular matrix protein whose downregulation is related to diminished expression of the main markers of epidermal differentiation [42]. Its secretion by the MSCs plays an important role in the remodeling of the lymphatic vasculature, which is essential for immune surveillance [43]. SULT1A1 is involved in sulphonation, a defense mechanism that eliminates certain chemicals from the body, such as some drugs, hormones, neurotransmitters, or xenobiotic compounds. The lack of this protein has been related to several hepatic pathologies in humans [44]. Finally, the complement component 4 binding protein alpha (C4BPA) participates mainly in inflammatory processes and in the metabolism of lipids [45].

When quantifying the expression of the proteins that were present in the secretomes from both groups, we found several alterations in their proteomic profiles. Here, 56 proteins were overexpressed by the cells from the diseased cats, and these proteins were mainly related to metabolic processes, inflammation, immune responses, oxidative stress, and angiogenesis. The FCGS group’s secretome also had 9 proteins whose expression was downregulated, which were involved in immune responses, mitochondrial activity, and cell adhesion.

The MSCs have been demonstrated to be more resistant to viral infections, such as dengue, Ebola, SARS, and influenza viruses, compared to differentiated cells [46,47]. This property has been related to the activation of genes stimulated by IFN, preventing the viruses from trespassing into the cell membrane, blocking gene transcription, protein synthesis, and the production of new viruses [48,49]. Nevertheless, most of the mechanisms used by the FCV to avoid the immune reaction of the host and to alter cell functions are still largely unknown [50]. In this context, proteomic analyses can help us gain insight into these processes by identifying alterations in the proteomic profiles. This study is a first step towards understanding the molecular basis of the FCV infection and how this affects the MSCs in FCGS patients. In previous work, we already showed that the proteomic profile of fAd-MSC-derived exosomes was altered in FCV-positive FCGS patients [20]. Hence, the viral infection seems to affect both the pool of soluble factors as well as the exosomes produced by the MSCs. Furthermore, considering the importance that cell therapy with MSCs has for the treatment of many immune-mediated pathologies in cats [23,51,52,53], and particularly in FCGS [9,54], these findings might help in evaluating the results of therapies based on the administration of autologous MSCs [55,56].

The present study has, however, some limitations, such as the number of cats that were included, the age differences between individuals, the little information available in the cat databases, and the aforementioned sensitivity of the multiplex assays, as well as the possible inter-individual variability of the secretomes. Therefore, although this work might set a base for understanding how FCV infection affects the behavior of MSCs, future studies are needed to unravel how FCV and other concomitant virosis influence the MSCs from other origins in cats with FCGS, as well as in FCV-positive cats that have not developed FCGS. These data will shed light on the question of whether the altered fAd-MSC secretome profile is due to gingivostomatitis, FCV infection, or a combination of both.

## 5. Conclusions

To our knowledge, this is the first study to compare the proteomic profiles in the MSC-derived secretomes from healthy and FCV-positive FCGS patients, demonstrating that the pro-inflammatory cytokines IL-6, IL-8, KC, and RANTES are overexpressed by the cells from diseased cats. Furthermore, among 1030 proteins expressed by the fAd-MSCs, the studied condition causes the overexpression of 56 proteins and the opposite for the other 9 proteins. These proteins are related to functions such as metabolic processes, immune responses and inflammation, homeostasis and protein folding, cell movement and interaction, oxidative stress, angiogenesis, mitochondrial metabolism, and cell adhesion. Three proteins were found exclusively in the secretome of FCGS patients (SVEP1, SULT1A1, and C4PBA), while three others (ALDOB, CRMP1, and SAA) could not be found in this sample. These proteins are mainly related to metabolic processes and immune regulation. All of these data together suggest that fAd-MSCs isolated from FCV-positive cats with FCGS produce a secretome with an altered proteomic profile.

## Figures and Tables

**Table 1:** Sex, age, weight, and SDAI scores of the cats selected for this study. The animals were the same as those previously used to analyze the protein profiles of exosomes produced by their fAd-MSCs [20].

Group	Sex	Weight (kg)	Age (Years)	SDAI Score
FCGS patients	♂	2.9	2.5	23
	♂	3.3	11.5	23
	♂	3.4	9	22
	♀	3.1	4	23
	♀	4.5	5	21
	Average	3.4 ± 0.6	6.4 ± 3.7	22.4 ± 0.9
Healthy cats	♂	4.1	3	
	♂	4	4	
	♂	3.8	6	
	♀	3.7	3	
	♀	3.5	5	
	Average	3.8 ± 0.24	4.2 ± 1.3	

## Data Availability

The data presented in this study are available in this published article (and its Supplementary Materials).

## Supplementary Materials

The following supporting information can be downloaded at: https://www.mdpi.com/article/10.3390/v14061146/s1. Figure S1: Characterization of fAd-MSCs. Table S1: List of total proteins with upregulated, downregulated, and exclusive proteins in the secretomes of the FCGS group and healthy donors. Table S2: Legends for upregulated and downregulated proteins according to the STRING database.
