# Supplementary material for: Secretory Profile of Adipose-Tissue-Derived Mesenchymal Stem Cells from Cats with Calicivirus-Positive Severe Chronic Gingivostomatitis
Source: Viruses. 2022 May 25;14(6):1146. doi: 10.3390/v14061146 (PMC9228153; doi:10.3390/v14061146)
Supplement: Supplementary file 1 [file viruses-14-01146-s001.zip › Figure S1_Assessment of adipogenic, osteogenic anf chondrogenic differentiation.pdf]

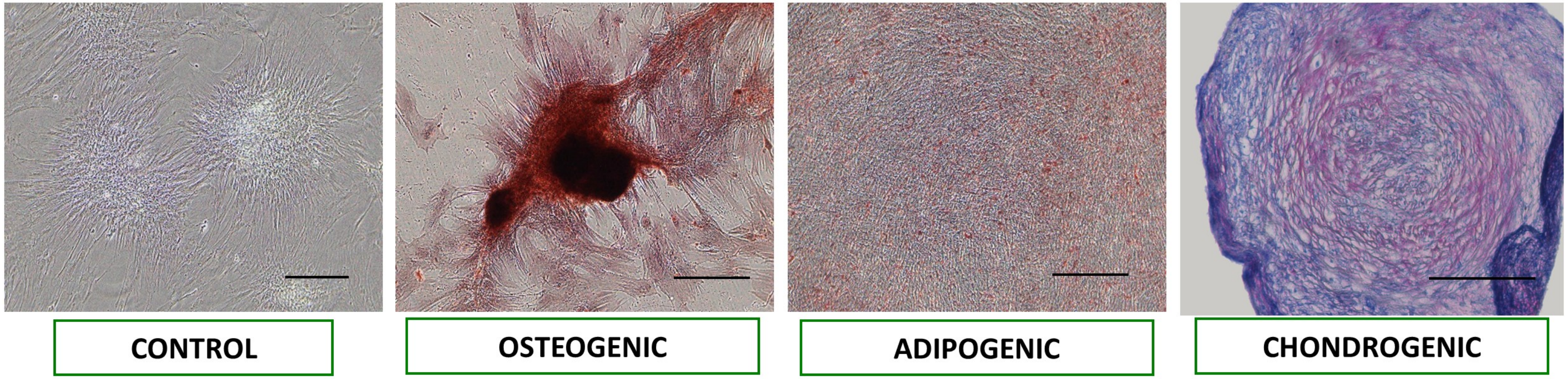

Figure S1. Assessment of adipogenic, osteogenic anf chondrogenic differentiation. Osteogenic differentiation is positive for alizarin red S. Adipogenic differentiation is positive for oil red O and chondrogenic differentiation is positive for toluidine blue. Bars, 200 μm.
